# D-dimer in Routine Hospital Practice: Diagnostic Accuracy for Venous Thromboembolism

**DOI:** 10.7759/cureus.117521

**Published:** 2026-10-06

**Authors:** Ammour Abdesselam, Reda Amrani Souhli, Moussab Arbai, Mouna Houari

**Affiliations:** 1 Medical Biology Department, Faculty of Medicine and Pharmacy, Abdelmalek Essaadi University, Tangier, MAR; 2 Hematology and Immunohematology Laboratory, Mohammed VI University Hospital Center, Tangier, MAR

**Keywords:** d-dimer, diagnostic performance, negative predictive value, pulmonary embolism, venous thromboembolism

## Abstract

Background

D-dimer is a sensitive rule-out test for venous thromboembolism (VTE), but most validation data come from selected cohorts with suspected embolism, and its performance in unselected hospital populations is less well characterized. We assessed the diagnostic performance of plasma D-dimer for VTE in an unselected hospital population, with pulmonary embolism (PE) as a subgroup, and evaluated the impact of an age-adjusted D-dimer threshold.

Methods

In this one-year retrospective study (May 2025-April 2026), 100 patients were included from 143 consecutive plasma D-dimer requests at a tertiary university hospital in Tangier, Morocco, irrespective of department or clinical indication. D-dimer was measured by immunoturbidimetric assay (threshold 500 ng/mL) and compared with CT pulmonary angiography (CTPA) for PE and venous Doppler ultrasound for deep vein thrombosis (DVT) in patients with a documented reference standard, with PE as a pre-specified subgroup.

Results

The study population (53% female; mean age 55.6 years) had D-dimer requested across many departments, most often adult emergency (54%). Values were right-skewed (median 814 ng/mL); 65% were positive. Imaging confirmed thromboembolic disease in 15 patients, including 12 venous events (nine PE, three DVT), and excluded it in 26. For VTE, sensitivity was 91.7% (95% CI 61.5-99.8) and negative predictive value (NPV) 92.9% (95% CI 66.1-99.8), with specificity 50.0% (95% CI 29.9-70.1), positive predictive value (PPV) 45.8% (95% CI 25.6-67.2) and an area under the curve (AUC) of 0.72 (95% CI 0.55-0.87). For PE, sensitivity and NPV both reached 100% (95% CI 66.4-100.0 and 73.5-100.0), with specificity 52.2% (95% CI 30.6-73.2). An age-adjusted D-dimer threshold raised specificity for VTE to 65.4% without loss of sensitivity.

Conclusion

In this unselected hospital population, plasma D-dimer showed high sensitivity for PE but low specificity, in keeping with its established role as a rule-out test. Its use in routine practice should be guided by clinical pretest probability and, where appropriate, age-adjusted thresholds.

## Introduction

Venous thromboembolism (VTE) ranks among the commonest vascular causes of death and weighs heavily on health systems [1]. Its non-specific clinical presentation relies principally on objective diagnostic testing. CT pulmonary angiography (CTPA) remains the reference imaging modality, but its radiation and contrast-related hazards make it valuable to identify in advance which patients truly need it [2]. Current guidelines, therefore, recommend a sequential strategy that pairs a structured estimate of clinical probability with D-dimer measurement and reserves imaging for patients who cannot be cleared otherwise [2]. D-dimer is well established for excluding VTE when pretest probability is low or moderate [3], whereas clinical probability itself is graded with validated rules such as the Wells score [4], the revised Geneva score [5], and the pulmonary embolism (PE) rule-out criteria [6]. More recent algorithms, including YEARS [7] and PEGeD [8], have refined the approach by tying the D-dimer cut-off to the pretest probability.

D-dimer is a degradation product of cross-linked fibrin that is released whenever the coagulation and fibrinolytic systems are activated. A result below the assay cut-off reflects negligible fibrin turnover and argues against significant thrombosis, whereas a higher value may signal an underlying clot, with greater concentrations broadly reflecting a larger thrombotic burden [3]. Diagnostic performance nonetheless varies with the method used. Quantitative enzyme-linked immunosorbent assay (ELISA), immunoturbidimetric, and fluorescence assays favor sensitivity over specificity, whereas whole-blood agglutination tests are more specific but less sensitive. The marker is also intrinsically non-specific, since concentrations rise in malignancy, infection, pregnancy, recent surgery or trauma, inflammation, disseminated intravascular coagulation, and renal impairment [9]. For this reason, an elevated D-dimer is not used to rule in thromboembolism, given its low specificity and positive predictive value (PPV). Because concentrations also increase with age, age-adjusted thresholds have been derived and prospectively validated to preserve specificity in older patients without compromising safety [10].

Most of the evidence supporting these thresholds and algorithms derives from selected cohorts of patients with clinically suspected PE, frequently in emergency or outpatient settings, and is reflected in current diagnostic guidelines [2]. In routine hospital practice, by contrast, D-dimer is requested far more broadly, across many departments and for indications that extend well beyond suspected thromboembolism, and comparatively little is known about its specificity and PPV in such unselected inpatient and emergency populations, particularly in our setting. The primary objective of this study was to assess the diagnostic performance of plasma D-dimer for VTE in an unselected hospital population. Secondary objectives were to evaluate D-dimer performance specifically for PE, describe the demographic and clinical characteristics of patients undergoing D-dimer testing and requesting patterns across hospital departments, assess the relationship between D-dimer levels and thromboembolic events, and evaluate the impact of an age-adjusted D-dimer threshold on diagnostic performance.

## Materials and methods

Study design

This was a retrospective diagnostic performance study conducted over a one-year period, from May 2025 to April 2026, in the Hematology laboratory of Mohammed VI General University Hospital (GUH) in Tangier, Morocco.

Study population and types of specimens

Of the 143 consecutive D-dimer requests received during the study period, 43 were excluded because of insufficient sample volume (non-conforming specimen, n = 17), incomplete laboratory or clinical data (n = 15), or duplicate entries for the same patient (n = 11), leaving 100 specimens for inclusion. Specimens were collected from patients hospitalized at Mohammed VI GUH for whom a plasma D-dimer assay had been requested as part of routine clinical care, irrespective of the requesting department or clinical indication. Only the first valid request per patient was retained. D-dimer was measured on citrated venous plasma obtained from blood drawn into 3.2% sodium citrate tubes.

Data collection and variables studied

Data were retrieved from the laboratory information system and, where necessary, from the patients' clinical records. The variables collected included age, sex, requesting clinical department, presenting clinical signs, plasma D-dimer concentration, documented thromboembolic events, and available imaging findings.

Imaging investigations were performed as part of routine clinical care according to the treating physician's clinical assessment and were not mandated by the study protocol. Imaging was interpreted by radiologists as part of routine care, independently of this study.

D-dimer measurement

Blood samples were centrifuged at 3,000 rpm for 10 minutes, and plasma D-dimer testing was performed within two hours of sample collection. Plasma D-dimer was quantified by a latex-enhanced immunoturbidimetric assay on an ACL TOP 350 CTS analyzer (HemosIL D-Dimer HS 500, Werfen/Instrumentation Laboratory, Bedford, MA, USA), with results expressed in ng/mL fibrinogen-equivalent units (FEU). In line with the manufacturer's clinically validated threshold, a result was considered positive when it exceeded the cut-off of 500 ng/mL [11]. As a secondary criterion, an age-adjusted threshold was applied, defined as the patient's age multiplied by 10 ng/mL in patients older than 50 years [10].

Internal quality control was performed daily using HemosIL D-Dimer HS 500 Controls at two levels (level 1 near the clinical cut-off and level 2 in the abnormal range). Results were evaluated according to Westgard rules and accepted when within the manufacturer's lot-specific acceptance ranges. Controls were also analyzed after each calibration or reagent lot change. Calibration was performed using the HemosIL D-Dimer HS 500 Calibrator (Werfen/Instrumentation Laboratory, Bedford, MA, USA) according to the manufacturer's instructions.

Reference standard

Imaging-confirmed diagnoses served as the reference standard. VTE was defined as imaging-confirmed PE and/or DVT. PE was considered confirmed when CTPA reported an endoluminal filling defect within the pulmonary arterial tree, and excluded when CTPA was reported as negative. DVT was diagnosed based on venous Doppler ultrasound findings and excluded when the examination was reported as negative. Ischemic stroke (brain imaging) and limb ischemia (CT angiography) were considered arterial thrombotic events and were not included in the VTE outcome.

When no imaging report was available in the reviewed medical record, thromboembolic status was considered not documented rather than negative. These specimens were retained for descriptive analyses but excluded from the diagnostic-performance analyses. Because PE and DVT were confirmed or excluded by different reference tests, comparator groups were defined separately for each outcome: patients with a negative CTPA for PE, patients with a negative venous Doppler ultrasound for DVT, and the union of these two groups for VTE overall.

Statistical analysis

Data were entered using Microsoft Excel (Microsoft Corporation, Redmond, WA, USA) and analyzed with IBM SPSS Statistics software, version 26.0 (IBM Corporation, Armonk, NY, USA). Categorical variables were presented as frequencies and percentages, whereas quantitative variables were reported as means with standard deviations or as medians with interquartile ranges (IQRs) according to their distribution, which was assessed with the Shapiro-Wilk test. Comparisons of proportions were performed using Pearson's chi-square test or Fisher's exact test when the assumptions for the chi-square test were not met (expected cell count below five). Continuous variables were compared between groups with the Mann-Whitney U test, and the association between two continuous variables was assessed with Spearman's rank correlation coefficient, given the skewed distribution of D-dimer. The diagnostic performance of D-dimer was expressed, for VTE overall and for PE and DVT separately, as sensitivity, specificity, PPV, and negative predictive value (NPV), with exact binomial (Clopper-Pearson) 95% confidence intervals (CIs). Overall discriminative ability was assessed by the area under the receiver operating characteristic (ROC) curve, and the optimal cutoff was identified using the Youden index. Agreement between the dichotomized D-dimer result and the imaging reference standard was quantified by Cohen's kappa (κ) coefficient, with its 95% CI obtained by the normal approximation. A p-value below 0.05 was considered statistically significant. In addition, diagnostic performance was recalculated using an age-adjusted D-dimer threshold (age × 10 ng/mL for patients older than 50 years, and 500 ng/mL for patients aged 50 years or younger).

Ethical considerations

This retrospective study was performed on-site and was based exclusively on pre-existing anonymized laboratory data, with no additional sample collection, no direct patient contact, and no modification of patient management. It was conducted in accordance with the Declaration of Helsinki and received a posteriori approval from the Tangier University Hospital Ethics Committee (ref. no. 297/26) on 21 September 2026. In accordance with national regulations and the non-interventional nature of the study, the requirement for informed consent was waived by the ethics committee.

## Results

Study population

Over the study period, 100 patients who underwent plasma D-dimer testing were included in the analysis. The study population comprised 53 females (53.0%) and 47 males (47.0%), with a female-to-male ratio of 1.13. The mean age was 55.6 years (SD, 21.4 years; median, 62 years; range, three to 90 years). Adult patients (≥18 years) represented the large majority of the cohort (93.0%, 93/100), whereas pediatric patients accounted for 7.0% (7/100).

D-dimer requests most frequently originated from the adult emergency department (54.0%), followed by hematology (9.0%) and the intensive care units (8.0%). The most common presenting sign was dyspnea (34.0%), followed by chest pain (12.0%) and impaired consciousness (9.0%). The baseline demographic and clinical characteristics of the study population are summarized in Table 1.

**Table 1:** Patient demographics and clinical characteristics (n = 100)

Variable	n (%)
Sex	
Male	47 (47.0)
Female	53 (53.0)
Age group	
Pediatric (<18 years)	7 (7.0)
18–59 years	39 (39.0)
≥60 years	54 (54.0)
Requesting department	
Adult emergency	54 (54.0)
Hematology	9 (9.0)
Intensive care	8 (8.0)
Pneumology	4 (4.0)
Other departments	25 (25.0)
Clinical sign	
Dyspnea	34 (34.0)
Chest pain	12 (12.0)
Impaired consciousness	9 (9.0)
Chest pain + dyspnea	3 (3.0)
Other/not documented	42 (42.0)

Distribution of D-dimer values and confirmed thromboembolic events

Plasma D-dimer values were strongly right-skewed (skewness 3.9; Shapiro-Wilk p < 0.001), with a median of 814 ng/mL (IQR, 350-1342; range, 17-8296) (Figure 1). Using the 500 ng/mL threshold, 65 samples (65.0%) were classified as positive and 35 (35.0%) as negative. Thromboembolic disease was confirmed by imaging in 15 patients (15.0%), excluded in 26 (26.0%), and not documented in the remaining 59 (59.0%). Among the imaging-confirmed events, PE was the most frequent (9/15, 60.0%), followed by deep vein thrombosis (DVT; 3/15, 20.0%), ischemic stroke (2/15, 13.3%), and limb ischemia (1/15, 6.7%). The distribution of D-dimer results and confirmed events is presented in Table 2.

**Figure 1:**
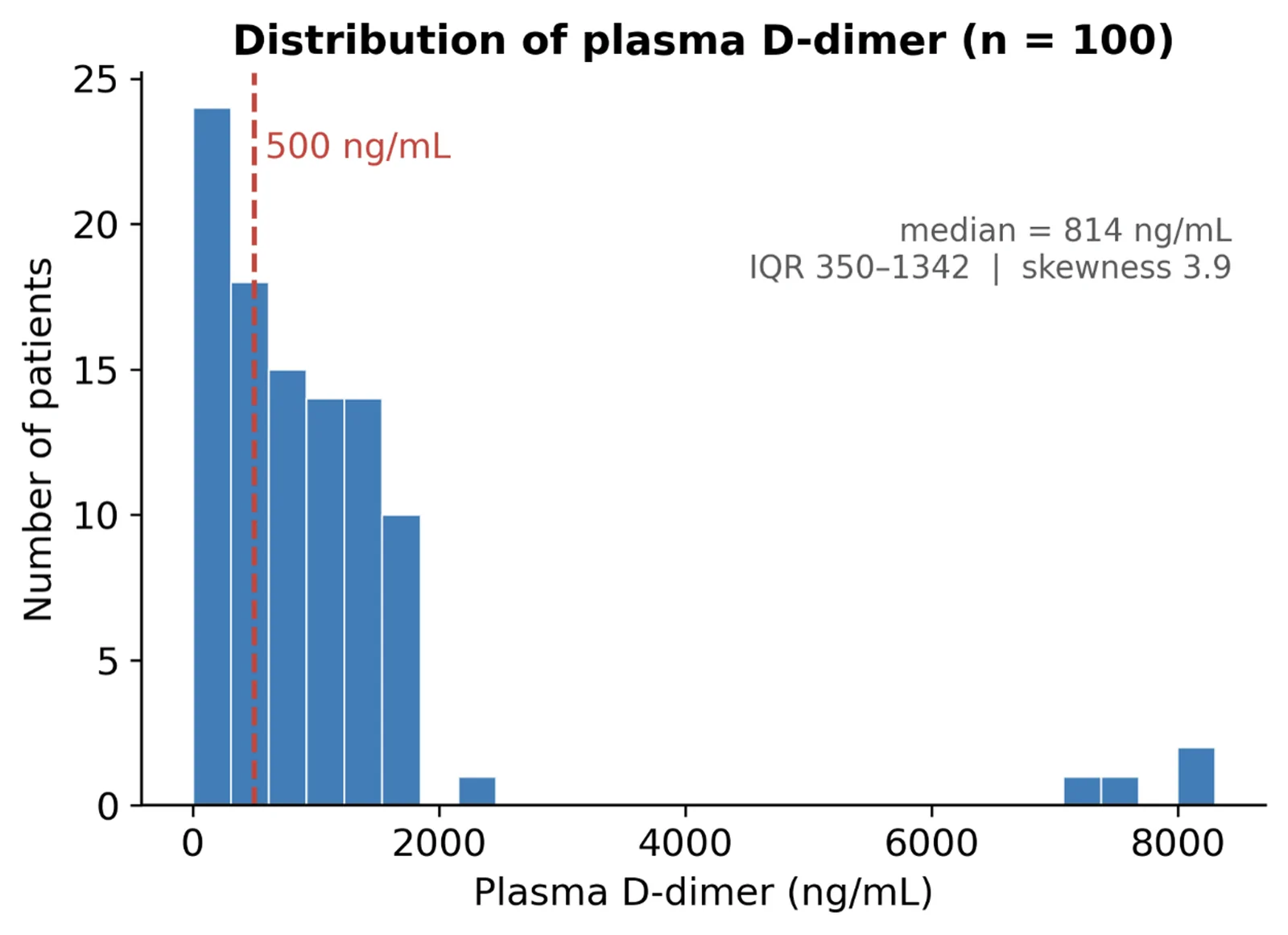
Distribution of plasma D-dimer in the study cohort (n = 100). The dashed line marks the 500 ng/mL threshold.

**Table 2:** Distribution of D-dimer results and imaging-confirmed events (n = 100) Percentages for confirmed events are expressed relative to the full cohort (n = 100); in the text, the same events are also given relative to the 15 confirmed cases.

Variable	n (%)
D-dimer result	
Positive	65 (65.0)
Negative	35 (35.0)
D-dimer result (age-adjusted threshold)	
Positive	61 (61.0)
Negative	39 (39.0)
Thromboembolic status	
Confirmed	15 (15.0)
Excluded	26 (26.0)
Not documented	59 (59.0)
Confirmed event on imaging	
Pulmonary embolism	9 (9.0)
Deep vein thrombosis	3 (3.0)
Ischemic stroke	2 (2.0)
Limb ischemia	1 (1.0)

Association of D-dimer with thromboembolic disease

The association between D-dimer and thromboembolic disease was assessed in the 41 patients with a determined status. A positive D-dimer was found in 12 of 15 patients with confirmed disease and in 15 of 26 without. The association did not reach statistical significance (Fisher exact test, p = 0.186), and D-dimer concentrations, although numerically higher in patients with disease (median 1072 vs 731 ng/mL), did not differ significantly (Mann-Whitney U test, p = 0.213).

Age, sex, and imaging correlates

Neither age nor sex was associated with the occurrence of thromboembolic disease. Age did not differ between affected and unaffected patients (median 63 vs 62 years; Mann-Whitney p = 0.745), and dichotomization at 50 years showed no relationship (Fisher exact p = 1.000). Sex was likewise not associated with disease (Fisher exact p = 0.754). Plasma D-dimer concentration was, however, weakly but significantly correlated with age (Spearman ρ = 0.24, p = 0.017). In contrast, D-dimer positivity was significantly associated with PE on imaging (Fisher exact p = 0.017): all nine patients with PE had an elevated D-dimer, and no embolism was observed below the threshold.

Diagnostic performance of D-dimer for VTE

Of the 100 patients, 41 had documented imaging (15 with confirmed thromboembolic disease and 26 with disease excluded). The three arterial events (two ischemic strokes and one limb ischemia) were also excluded, as D-dimer is validated exclusively for VTE, restricting the analysis to the 12 venous cases (nine PE, three DVT). Because PE and DVT were confirmed or excluded by different reference tests, comparator groups were defined separately for each outcome.

Against this comparator, D-dimer at 500 ng/mL achieved, for VTE overall, a sensitivity of 91.7% (95% CI, 61.5-99.8) and a NPV of 92.9% (95% CI, 66.1-99.8), with a specificity of 50.0% (95% CI, 29.9-70.1) and a PPV of 45.8% (95% CI, 25.6-67.2). Discriminative ability was assessed by ROC analysis (area under the curve (AUC) 0.72, 95% CI 0.55-0.87), with a Youden-optimal cutoff of 820 ng/mL (Figure 2). Performance was highest for PE, where all nine confirmed cases were D-dimer positive (sensitivity and NPV both 100%; specificity 52.2%, 95% CI 30.6-73.2; PPV 45.0%, 95% CI 23.1-68.5); the single false-negative was a DVT below the threshold. Agreement with the reference standard was fair for PE (κ = 0.38) and for VTE overall (κ = 0.33), and could not be reliably estimated for DVT alone given the small subgroup (n = 6, κ = 0.00). These results are summarized in Table 3.

**Figure 2:**
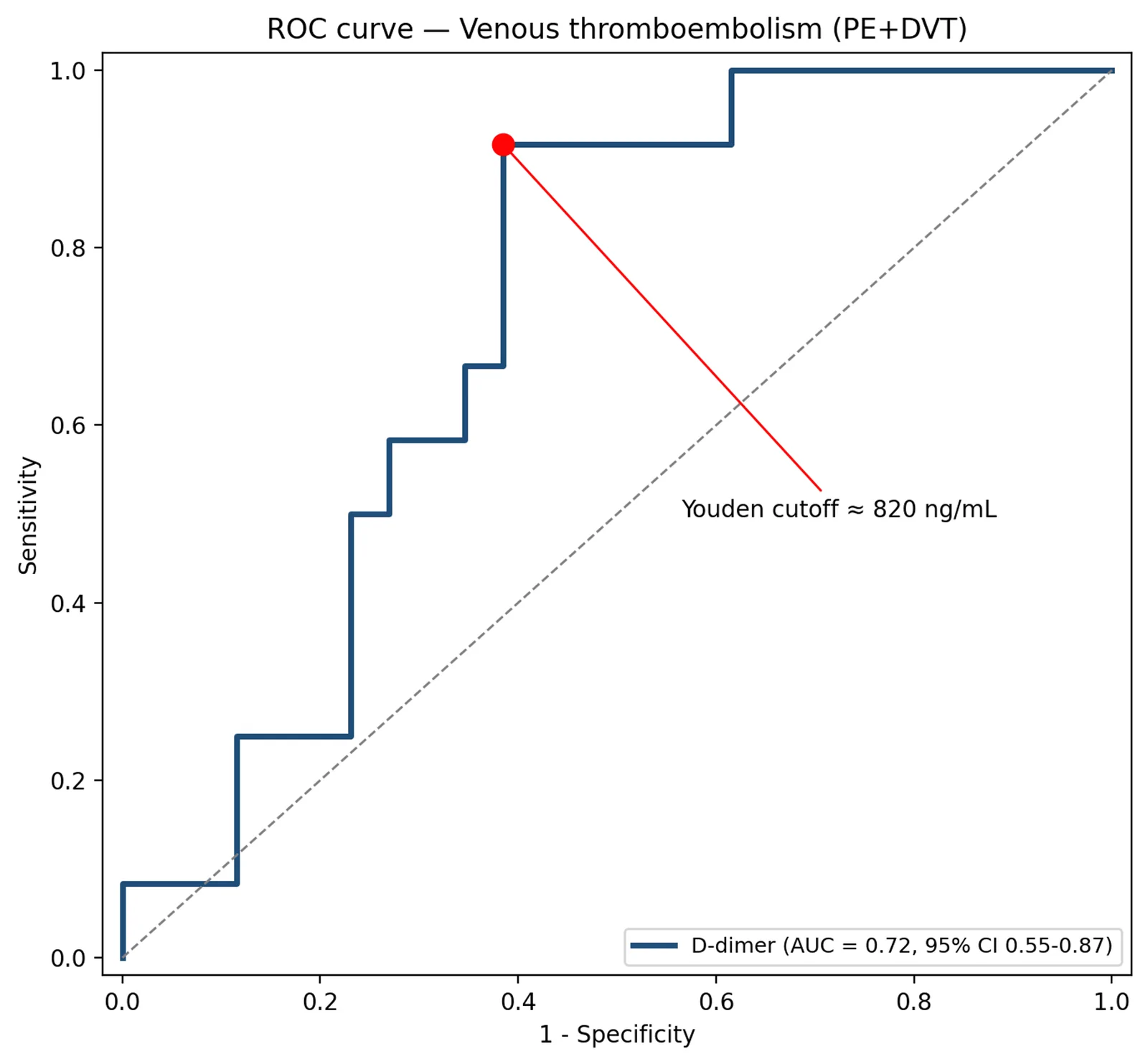
Receiver-operating-characteristic (ROC) curve of plasma D-dimer for venous thromboembolism (12 confirmed cases vs. 26 outcome-specific imaging-negative comparators). The area under the curve (AUC) was 0.72 (95% CI, 0.55–0.87); the marked point indicates the Youden-optimal threshold (≈820 ng/mL). Abbreviations: CI, confidence interval; PE, pulmonary embolism; DVT, deep vein thrombosis

**Table 3:** Diagnostic performance of plasma D-dimer (500 ng/mL threshold) for VTE, against outcome-specific comparators in whom the corresponding reference test excluded disease. Abbreviations: CI, confidence interval; PPV, positive predictive value; NPV, negative predictive value; PE, pulmonary embolism; DVT, deep vein thrombosis; VTE, venous thromboembolism Because PE and DVT were confirmed or excluded by different reference tests, comparator groups were defined separately for each outcome: the 23 patients with a negative CTPA served as the comparator for PE, and the 3 patients with a negative venous Doppler ultrasound served as the comparator for DVT; the combined VTE analysis used the union of these two outcome-appropriate comparator sets (26 patients in total). Confidence intervals for sensitivity, specificity and predictive values are exact binomial (Clopper–Pearson) intervals.

Outcome	Cases (+/−)	Sensitivity % (95% CI)	Specificity % (95% CI)	PPV % (95% CI)	NPV % (95% CI)
PE	9/23	100.0 (66.4–100.0)	52.2 (30.6–73.2)	45.0 (23.1–68.5)	100.0 (73.5–100.0)
DVT	3/3	66.7 (9.4–99.2)	33.3 (0.8–90.6)	50.0 (6.8–93.2)	50.0 (1.3–98.7)
VTE (PE+DVT)	12/26	91.7 (61.5–99.8)	50.0 (29.9–70.1)	45.8 (25.6–67.2)	92.9 (66.1–99.8)

When an age-adjusted D-dimer threshold (age × 10 ng/mL for patients aged ≥50 years) was applied instead of the fixed 500 ng/mL cut-off, four D-dimer-positive, CTPA-negative comparators among the PE comparator group were reclassified as negative, without reclassifying any confirmed venous event. For VTE overall, specificity increased from 50.0% to 65.4% (95% CI, 44.3-82.8) and PPV from 45.8% to 55.0% (95% CI, 31.5-76.9), while sensitivity remained at 91.7% and NPV rose slightly to 94.4% (95% CI, 72.7-99.9). For PE specifically, specificity increased from 52.2% to 69.6% (95% CI, 47.1-86.8) and PPV from 45.0% to 56.2% (95% CI, 29.9-80.2), while sensitivity and NPV remained at 100%. The DVT-specific analysis was unaffected.

## Discussion

In this retrospective study, plasma D-dimer was measured in a predominantly adult population (93.0% aged ≥18 years), with a near-balanced sex distribution (female-to-male ratio 1.13) and a mean age of 55.6 years. The test was requested across a wide spectrum of departments. This pattern confirms that, in routine hospital practice, D-dimer is ordered well beyond the strict context of suspected VTE, a usage that has direct implications for the test’s specificity discussed below [12].

Consistent with this broad use, D-dimer was strongly right-skewed, and 65.0% of samples exceeded the 500 ng/mL threshold, whereas PE was ultimately confirmed in only nine patients. The high positivity rate therefore reflects the heterogeneous case mix and the diversity of indications rather than a high prevalence of thrombosis and indicates that an elevated value in this setting should be regarded as a finding that prompts, rather than replaces, definitive evaluation [9].

The association between D-dimer and thromboembolic disease differed according to how the outcome was defined. D-dimer was significantly associated with PE specifically (p = 0.017) but not with the broader category of any thromboembolic event (p = 0.186). The loss of signal in the broad analysis was driven by arterial events such as ischemic stroke and limb ischemia, in which D-dimer is not a validated marker. This supports restricting the diagnostic application of D-dimer to VTE and interpreting it within a specific clinical question, in line with the VTE focus of current guidelines [2,3].

In our study, neither age nor sex was associated with the occurrence of thromboembolic disease, yet plasma D-dimer concentration was itself weakly but significantly correlated with age (Spearman ρ = 0.24, p = 0.017). This is in keeping with the well-described age-related rise in baseline D-dimer, whereby the specificity of the test falls progressively with age, so that a normal result excludes embolism in a far smaller proportion of older than younger patients [13]. This age-related loss of specificity underpins the age-adjusted threshold (age × 10 ng/mL for patients aged 50 years or older), derived from large retrospective cohorts [14], supported by meta-analysis [15], and prospectively validated in the ADJUST-PE study, in which combining clinical probability with an age-adjusted cutoff increased the proportion of patients in whom embolism could be excluded while maintaining a three-month failure rate of only 0.3% [10]. Applying such a threshold to our population would be expected to improve specificity without materially compromising sensitivity.

The high sensitivity and NPV we observed are consistent with the large body of evidence supporting D-dimer as a rule-out test. Its high sensitivity and low specificity yield a high NPV that makes it useful to exclude VTE in the appropriate clinical setting [16], and this property has been operationalized in validated management strategies. The Christopher study showed that a dichotomized Wells score combined with D-dimer could safely exclude PE without imaging in a substantial proportion of patients [17], and subsequent algorithms such as YEARS and the PEGeD strategy have refined this approach using variable D-dimer thresholds according to clinical probability, including adaptations for special populations such as pregnancy [7,8,18]. In the YEARS study, PE was excluded when no clinical items were present, and D-dimer was below 1000 ng/mL, or when one or more items were present, and D-dimer was below 500 ng/mL, reducing CT angiography by an absolute 14% [7]; varying the threshold according to pretest probability has likewise been shown to increase specificity while preserving an NPV above 99% [8]. The sensitivity of 91.7% for VTE overall, reaching 100% for PE, in which all confirmed cases were D-dimer-positive, is concordant with these data, though it must be read against our small number of events.

The low specificity and PPV we found are equally consistent with the literature and are clinically the most important caveat. Because fibrin turnover is increased in a wide range of conditions, including cancer, infection, and tissue necrosis, the PPV of D-dimer for PE is low [19], and pregnancy, trauma, malignancy, surgery, and liver disease all raise D-dimer concentrations [16]. Our study was dominated by inpatients and emergency presentations across many specialties, in whom D-dimer was often requested outside the strict context of suspected embolism, making a high false-positive rate inevitable. Diagnostic studies and systematic reviews using imaging as the reference have reported specificities of roughly 40%-50% at the 500 ng/mL cutoff [3,20], and the increasing, low-yield use of D-dimer and CT angiography has been linked to the overdiagnosis of subsegmental embolism, radiation exposure, and contrast-related risk [7,21]. The fair kappa in our study quantifies this limitation directly.

Limitations

Several limitations should be acknowledged. The retrospective, single-center design and modest sample size limit generalizability, and the small number of confirmed PE produced wide CI and an absence of false negatives that should be interpreted cautiously rather than as proof of perfect sensitivity. Imaging was applied only to patients in whom it was clinically indicated, introducing verification and selection bias; thromboembolic status was undocumented in a large proportion of patients, who were necessarily excluded from the diagnostic analysis. The comparator patients were themselves selected for imaging by their treating physicians, so specificity and predictive values cannot be generalized to all patients tested. Structured pretest probability scores (Wells or revised Geneva), which are integral to the appropriate use of D-dimer, were not available, and inter-assay variability further limits direct comparison with cohorts using other methods.

## Conclusions

In this unselected hospital population, plasma D-dimer was highly sensitive for PE, in keeping with its established role as a rule-out test, whereas a raised value is non-specific. D-dimer should, therefore be interpreted in the context of clinical pretest probability and, where appropriate, age-adjusted thresholds. Larger prospective studies with systematic imaging and protocol-based pretest probability assessment are needed to confirm these findings and to refine the diagnostic performance of D-dimer in routine hospital practice.
